# Peptidomics analysis of plasma in patients with ankylosing spondylitis

**DOI:** 10.3389/fimmu.2023.1104351

**Published:** 2023-01-31

**Authors:** Guo-ning Zhang, Ying-jia Xu, Lei Jin

**Affiliations:** ^1^ Department of Orthopedics, Tongren Hospital, Shanghai Jiao Tong University School of Medicine, Shanghai, China; ^2^ Department of Laboratory Medicine, Tongren Hospital, Shanghai Jiao Tong University School of Medicine, Shanghai, China; ^3^ Department of Rheumatology and Immunology, Tongren Hospital, Shanghai Jiao Tong University School of Medicine, Shanghai, China

**Keywords:** ankylosing spondylitis, peptidomics, LC-MS/MS, CCK8, edu

## Abstract

**Background:**

This study aimed to explore the differential expression of peptides associated with ankylosing spondylitis (AS) patients, enabling identification of potential functional peptides to provide the basis for the novel intervention targets for AS.

**Material and Methods:**

3 AS patients and 3 healthy volunteers were enrolled in this study. The expression profiles for peptides present in the plasma of AS patients and the healthy individual were analyzed by liquid chromatography-tandem mass spectrometry (LC‐MS/MS). The physicochemical properties and biological functions of identified peptides were further analyzed by bioinformatics. The results of peptide identification were verified by cell viability analysis, using CCK8 and Edu staining assay, and the differential peptides relevant to the disease were screened.

**Results:**

52 differential peptides were successfully identified using mass spectrometry. 44 peptides were up-regulated, while eight were down-regulated. FGA-peptide (sequences: DSGEGDFLAEGGGVRGPR), C4A-peptide (sequences: NGFKSHAL), and TUBB-peptide (sequences: ISEQFTAMFR) were screened out that could significantly promote the proliferation of fibroblasts in AS patients. Bioinformatics analysis showed these differentially expressed peptides might be associated with “MHC class I protein binding” and “pathogenic Escherichia coli infection” pathways, which might further affect the progression of AS.

**Conclusion:**

This pilot study shows 3 differentially expressed peptides may have the potential function for the occurrence and development of AS, may provide novel insights into the underlying molecular mechanisms of AS based on peptide omics.

## 1 Introduction

Ankylosing spondylitis (AS) is one of the unsolved problems in the field of rheumatism, which finally results in bony ankylosis, discomfort, and impairment and mostly damages the lumbar spine as well as sacroiliac peripheral joints (1). As the disease progresses, the quality of life of AS patients gradually decreases. During the late stage, it might involve fusion of the spinal joint, sacroiliac joint, hip joint, and other joints. Consequently, the patients completely lose the ability to work and self‐care, adding a burden to society and patients’ families. AS is one of the most intractable diseases with high occurrence, a great risk of impairment, as well as a significant cost of care. Its root cause of the “three high” lies in our insufficient understanding of the occurrence and development of AS. Although the “arthritogenic peptide” theory has been proposed on the mechanism of HLA-B27 induced AS in recent years (2), and some scholars have also proposed that AS and HLA-B27 may be related to deficiency gut immunity (3), however, the exact pathogenesis of AS remains unclear, so far it has not been effectively treated. Therefore, it is still necessary to conduct in-depth research on the pathogenesis of AS, open new directions for the development of more effective therapeutic drugs and promote the final victory over AS.

Plasma is a source of biomarkers that reflects physiological and pathological conditions in the body. A growing number of studies are focused on proteins and peptides, including a number of studies conducted as part of the Human Proteome Project (HPP) of the Human Proteome Organization (HUPO). It is becoming increasingly apparent that proteomics and peptidomics techniques can be used in the development of novel preventative measures in precision medicine. With the advent of plasma proteomics and peptidomics, it has become possible to study the pathogenesis of diseases (e.g., COVID-19 and cancer) to identify valuable biomarkers and improve the clinical management of these diseases (4). Peptidomics is an emerging field of proteomics (5). In general, it is a method that is widely used for the assessment of liquid chromatography-tandem mass spectrometry (LC-MS/MS) for the detection of peptides contained in diverse biological materials (6, 7). It can be used for systematic, qualitative, and quantitative assessment of the composition and content of endogenous peptides occurring in organisms under physiological or pathological conditions. Developments in the peptidomics field assisted in the identification of a group of small‐molecule peptides of 3 to 50 amino acids that have a role in a number of biological processes, including cell differentiation (8), apoptosis (9), immune regulation (10), nervous system regulating (11), as well as reproduction regulation (12). It has been previously established that the variety and quantity of proteins and peptides mostly change even before the appearance of obvious symptoms or pathological changes of the disease (13). Therefore, the study of peptidomics exhibits great potential to explore the possible pathogenesis of various diseases.

In view of the effectiveness of peptideomics in exploring the underlying mechanisms of various diseases and few studies on peptideomics analysis related to AS so far, in this pilot study we perform peptidomic analysis of plasma in AS patients to explore the expressed peptides which may be involved in AS, could also provide fresh perceptions into the molecular pathways behind AS based on peptide omics.

## 2 Materials and methods

### 2.1 Subjects

A total of 6 subjects including 3 AS patients and 3 healthy volunteers were enrolled in this study. The patients with AS were recruited from the Orthopedic and Rheumatology Department at the Shanghai Tongren Hospital. The patients were required to meet the New York criteria for their inclusion (14). Patients with other chronic diseases such as hypertension or diabetes and those who have received treatment with a TNF inhibitor were excluded from this study. The Tongren Hospital’s Ethics Committee gave their approval to this research. (IRB: 2021-006-01). All subjects in this study provided their signed informed consent. Their peripheral blood was sampled and centrifuged (maximum time intervals between venepuncture and serum separation 1h); the supernatant plasma samples were immediately stored in the refrigerator at -80°C until further processing (15).

### 2.2 Sample processing steps

To the plasma samples (500 µL), -20°C of pre-cooled methanol was added in the ratio of 1:2. The mixture was vortexed at 4°C and precipitated for 1hr at periodically rotated every ten minutes. At 4°C, the resulting supernatant was centrifuged at 12000 xg for 20 min. Its resultant supernatant was collected and dried by freezing using a centrifugal concentrator. Then, ultrafiltration was used to remove more fluid and high-molecular-weight solutes. Phosphate-buffered saline (PBS) was added to dissolve the dried sample, and the solution was transferred to a new 10-kDa ultrafiltration tube (RT-UFC501096-5; Millipore). The ultrafiltration device was Spin at 10000 xg for 30 min at 4°C. Collect the filtrate. next, HiPPR-derivative removal spin column kit(Thermo Scientific, cat log: Thermo Scientific) and C18 micro columns (Thermo Scientific, cat log: 89870) were used to remove potential contamination for the following mass spectrometry analysis (16).

### 2.3 Identification of peptides using LC-MS/MS

The peptides were identified using the nanoLC-MS/MS on the Fusion Lumo (Thermo Fisher Scientific, Inc.) in combination with the EASY-nano-LC1200. For chromatographic separation, MilliQ water was mixed with 2 percent acetonitrile and 0.1 percent formic acid as solvent A buffer, and 90 percent acetonitrile and 0.1 percent formic acid as solvent B buffer. The plasma samples were reconstituted in 20 µL of solvent A, processed through the nano-LC for separation, and then subjected to an online electrospray tandem MS analysis. Onto the analytical column (75×250 µm; Acclaim PepMap C18; Thermo Fisher Scientific, Inc.), 8L of the peptide sample were loaded. The peptides were then eluted with 5% of solvent B for 5 minutes, 5-40% of solvent B for 65 minutes, 40-80% of solvent B for 1 minute, 80% of solvent B for 4 minutes, and 5% of solvent B more than 20 minutes at 300 nL/min. The MS spectra were collected with a mass resolution of 120K across the mass range of 350-2,000 m/z.

The sample was analyzed by on-line nanospray LC-MS/MS on an Thermo Scientific™ Orbitrap Fusion Lumos ™ coupled to an EASY-nano-LC 1200 system (Thermo Fisher Scientific, MA, USA). 5L peptide was loaded (analytical column: Acclaim PepMap C18, 75 pm x 25 cm) and separated with a 60 min linear gradient, from 6% B (B: 0.1% formic acid in 80% ACN) to 60% B. The column flow rate was maintained at 400 nL/min with the column temperature of 40°C. The electrospray voltage of 2 kV versus the inlet of the mass spectrometer was used.

The mass spectrometer was run under data dependent acquisition mode, and automatically switched between MS and MS/MS mode. The parameters were: (1) MS: scan range (m/z) = 100-1500; resolution=120,000; AGC target=4e5; maximum injection time=50ms; include charge states=1-7; (2) HCD-MS/MS: resolution=15,000; isolation window=3; AGC target=5e4; maximum injection time=35 ms; collision energy=30.

Peptides were identified using PEAKS (17) search program across the Swissprot_human database (https://www.uniprot.org/taxonomy/9606) with the following search parameters: monoisotopic parent mass tolerance of 10 ppm; fragment mass tolerance of 0.5 Da; modifications – oxidation of methionine; unspecific peptide cleavage. The result filters were set as PSM FDR <1% (16).

### 2.4 Bioinformatics analysis

Label-free quantification was used to determine the peptides’ intensity, whereas Peaks software was used to evaluate the MS/MS data. A fold change greater than 2 with P less than 0.05 (Student’s t-test) was the selection criteria for the differentially expressed peptides. The programme MetaboAnalyst 5.0 was used to create the heat map. Online calculators were used to determine the peptides’ molecular weight (MW) as well as isoelectric point (PI) (https://web.expasy.org/prot-param/). The UniProt database (http://www.uniport.org/) was used to evaluate the discovered peptides’ progenitor proteins. According to the “Molecular Function,” “Cellular Component,” and “Biological Process” subcategories of the Gene Ontology (GO) (18), the putative functions of the precursor proteins derived from the discovered peptides were examined GO as well as Kyoto Encyclopedia of Genes and Genomes (KEGG) pathways (19) utilizing DAVID Bioinformatics Resources 6.8’s Functional Annotation Tool (https://david.ncifcrf.gov). The protein interactions were analyzed using the STRING website (https://string-db.org/, version:11.0) and Cytoscape 3.5.1 software. The amino acid sequences of the different species were analyzed using the protein database on the NCBI website (https://www.ncbi.nlm.nih.gov/homologene/), and the results were compared with DNAMAN (version 9.0) software.

### 2.5 Screening and synthesis of peptides

We screened differential peptides according to the principles of high activity fraction of peptide ranker, large difference between groups and small difference within groups. The website also forecast the bioactivity of peptides(http://bioware.ucd.ie/), and the peptides with the top 10% activity were selected for preliminary functional evaluation. A total of 3 differential peptides were screened out. The physical and chemical properties were examined online using the website (https://web.expasy.org/protparam) and EMBL-EBI analysis (https://www.ebi.ac.uk/). These peptides were synthesized and the purity of peptides was >95%.

### 2.6 Cell viability analysis verification

#### 2.6.1 Cell culture

The fibroblasts in patients with AS were obtained from the ligamentum teres of the hip during total hip arthroplasty. The ligament tissue samples were collected from the operating table and immediately placed into a sterile bottle containing DMEM/F-12 medium, stored at 4°C, and primary cultured within 2 hr. First, PBS was used to clean the ligament tissue samples thrice to remove the blood and other components. After centrifugation for five minutes, The supernatant was eliminated, and two times as much DMEM/F-12 solution containing type-I collagenase was added and digested at 37°C for 4 hr. Next, 0.25% trypsin was added to the tissues for 15 minutes. Following digestion, the cells were filtered *via* a 200-mesh screen for collection, centrifuged at 800 rpm for five minutes, and the supernatant was discarded. The cells were suspended in DMEM/F12 media supplemented with 20% foetal bovine serum (FBS), 100 µg/M1 penicillin, and 100 g/M1 streptomycin. Initially, the cells were cultured for 24 hours at 37°C with 5 percent CO_2_ and saturated humidity after being injected at a density of 1×103/cm^2^ in a 25-cm2 plastic culture container. Afterwards, 5 mL of new media was added to wash off the suspended contaminants, blood cells, as well as non-adherent cells after seeing the ligament fibroblasts under a microscope. The media was then changed to DMEM/F-12 with penicillin and 10% foetal bovine serum. Under the microscope, ligament fibroblasts were observed to grow in a dense monolayer. Meanwhile, the cells were subcultured. After washing the cells with PBS, 2 mL of 0.25% trypsin solution was added; the cells were observed to have shrunk into a single round cell under the microscope. The cells were gently blown to suspend them, and then DMEM/F-12 medium supplemented with 10% foetal bovine serum was given. The supernatant was then removed from the cell solution by centrifuging it for five minutes at 800 rpm in a sterile 15-mL tube. A fresh medium was added to re-suspend the cells, and the cell concentration was adjusted to 3×103/cm^2^, after which the cells were inoculated into a new plastic cell culture bottle for further culture. In the experiment, the cells were subcultured in the ratio of 1:3. The third generation of fibroblasts in the patients with AS was used in this study.

#### 2.6.2 CCK-8 assay

Fibroblasts from the AS patients were plated in a 96-well plate at a density of 1 × 104 cells per well and grown at 37°C in a 5 percent CO_2_ environment. Cells were introduced at 70–80% confluency with varying amounts of the three produced peptides (50 μmmol/L), 5 compound wells per concentration,and each group was grown for 24 hours at 37°C with 5% CO_2_. In the control group, no peptide was introduced. 10 μL CCK-8 (Beyotime) reagent was then used, and the plate was cultured for an additional 4 hours after that. To determine the cell viability, the absorbance was measured at 450 nm using a Tecan Infinite M1000 Pro microplate reader (San Jose, CA, US). Cell viability = (absorbance of experimental wells - absorbance of blank wells)/(absorbance of control wells - absorbance of blank wells)×100%. The experiment was repeated three times.

#### 2.6.3 Edu staining assay

The fibroblasts in each group were inoculated into a 24-well plate with approximately 0.5×10^5^ cells/well and cultured overnight. The cells were treated with 50 μmmol/L peptides the next day in a biosafety cabinet. The cells were washed three times with sterile 1×PBS after the cell culture supernatant was removed and discarded. The diluted peptides were added at the rate of 100 uL/well. No peptide was added in the control group. The proliferation was detected by Edu immunofluorescence staining at 24 hr. Cell proliferation rate = number of proliferating cells/total number of cells × 100%.

### 2.7 Statistical analysis

Every piece of data was represented as mean standard deviation (SD). To examine statistical differences, either a one-way analysis of variance (ANOVA) with Bonferroni’s adjustment for multiple comparisons or an unpaired two-sided Student’s t-test were used. The threshold for statistical significance was fixed at P less than 0.05.

## 3 Results

### 3.1 Process followed for peptidomics analysis

**Figure 1:**
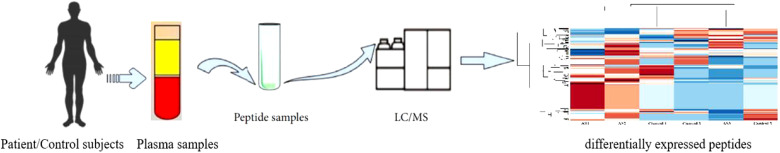
The process of peptide identificationin peripheral blood in patients with ankylosing spondylitis by LC/MS mass apectrometry.

For peptidomics analysis, the plasma samples were collected from AS patients and healthy volunteers. The individuals were all of the same age. (30–40 years). All these subjects were male and presented no previous history of any other chronic diseases. The clinical characteristics of the subjects were shown in **Table 1**. Using LC-MS/MS, the expression patterns of peptides found in the plasma of AS patients and a healthy person was compared. In **Figure 1**, the schematic procedure can be seen.

**Table 1:** The clinical characteristics of the subjects.

	Patient 1	Patient 2	Patient 3	Normal subject 1	Normal subject 2	Normal subject 3
Gender	Male	Male	Male	Male	Male	Male
Age	34	36	33	33	36	34
Body mass index (Kg/m^2^)	23.56	24.78	25.36	24.33	25.47	22.98
Disease duration (years)	2	1	2			
Blood routine leukocyte(g/L)	10.77×10^9^	11.84×10^9^	13.76×10^9^			
Erythrocyte sedimentation rate(mm/h)	10	18	28			
C-Reactive Protein (mg/L)	15.18	22.25	16.14			
Bath Ankylosing Spondylitis Disease Activity Index (BASDAI)	4.9	4.5	4.8			
Bath Ankylosing Spondylitis Functional Index (BASFI)	4.1	4.9	4.4			
Visual analogue scale (VAS)	5	6	5			

### 3.2 Identification of peptide expression profiles

**Figure 2:**
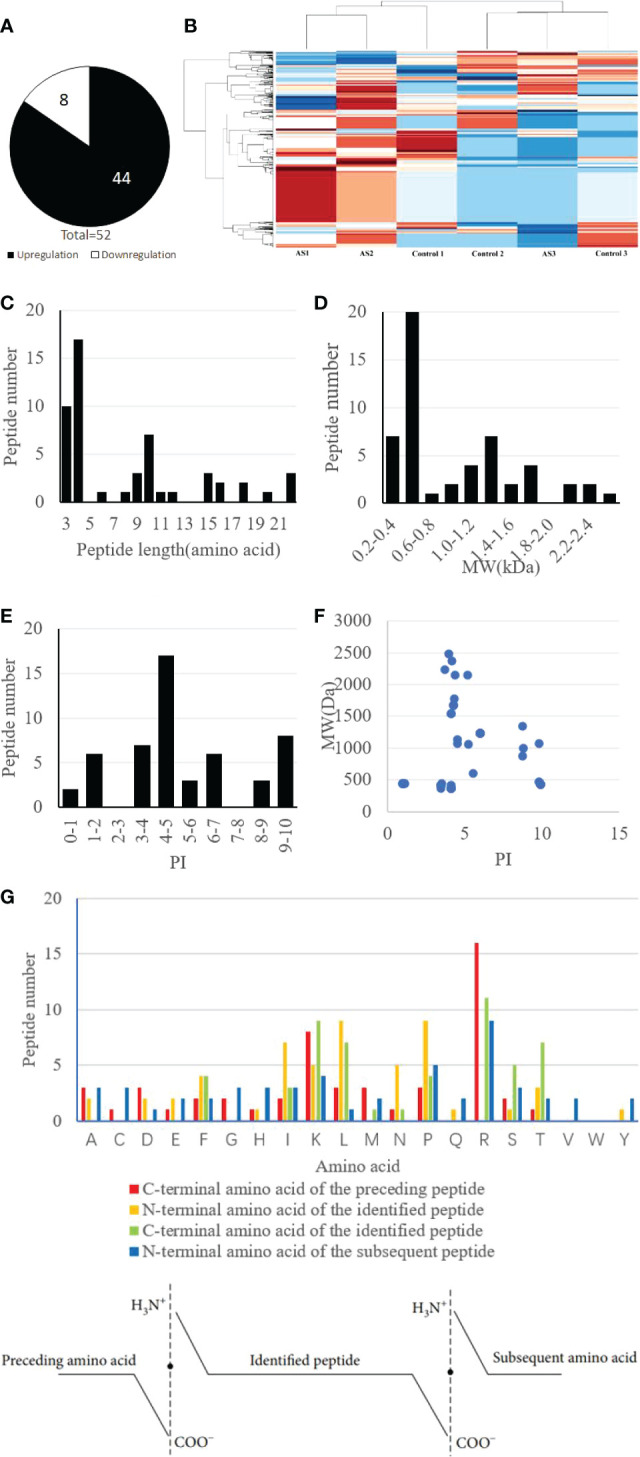
Identification and features of differentially expressed peptides. **(A)** Identification of the number of differentially expressed peptides. **(B)** Heat map of the differentially expressed peptides. **(C)** Distribution of the differentially expressed peptides by length. **(D)** Molecular weight (MW) of the differentially expressed peptides. **(E)** Isoelectric point (PI) of the differentially expressed peptides. **(F)** The correlation between the distribution of differentially expressed peptides by MW and PI. **(G)** Four cleavage sites of the differentially expressed peptides.

The results of MS analysis detected a total of 1559 peptides. Among these, 52 peptides exhibited differential expression in AS patients, against the control group (P less than 0.05 and fold change ≥2) (**Table 2**). Eight peptides were downregulated, whereas 44 were increased (**Figure 2A**). The heat map showed that the peptide profiles of AS patients and healthy people were significantly different from one another (**Figure 2B**). The distribution of 52 peptides in terms of length of the peptide revealed that these peptides were mainly concentrated in the range of 3–12 amino acids (**Figure 2C**). MW of these peptides was recorded to be in the range of 0.2–1.8 kDa, as well as PI scores were found to be between 3 and 10 (**Figures 2D, E**). Differentially expressed peptides’ MW and PI distributions were correlated, and this connection showed that these peptides were mostly concentrated into four groups, particularly close to PIs 4, 5, 6, 9, and 10. (**Figure 2F**).

**Table 2:** The details of the 52 differentially peptides (AS group VS Control group).

Protein names	Protein Accession	Peptide sequence	Fold change	*P* Value*
Albumin	P02768	T.FTF.H	+7.093558743	0.02097
Collagen alpha-1	Q07092	G.NSGEKGDQGFQGQPGFPGPPGP.P	-4.311473151	0.039273
Ubiquitin-activating enzyme E1	P22314	L.KATL.P	-7.256112727	0.007996
Ubiquitin-activating enzyme E1	P22314	N.FAM.I	+3.223158229	0.027864
Nischarin	Q9Y2I1	L.PFT.C	+3.22315836	0.027864
Putative elongation factor 1-alpha-like 3	Q5VTE0	M.LEPS.A	+3.22315836	0.027864
Elongation factor 1-alpha 1	P68104	M.LEPS.A	+3.22315836	0.027864
Alpha-1 type I collagen	P02452	D.TTLK.S	+3.223158464	0.027864
Proline-rich protein 4	Q16378	D.FTF.T	+7.093558743	0.02097
Caveolae-associated protein 1	Q6NZI2	P.PFT.F	+3.22315836	0.027864
Caveolae-associated protein 1	Q6NZI2	P.FTF.H	+7.093558743	0.02097
Alpha-2-HS-glycoprotein	P02765	C.KATL.S	-7.256112727	0.007996
Alpha-actinin-1	P12814	F.KATL.P	-7.256112727	0.007996
Alpha-actinin-1	P12814	K.LVSIGAEEIVDGNVK.M	+3.223158453	0.027864
Alpha-actinin-2	P35609	F.KATL.P	-7.256112727	0.007996
Alpha-actinin-2	P35609	K.LVSIGAEEIVDGNVK.M	+3.223158453	0.027864
Myosin-6	P13533	R.LEEAGGATSVQIEMNK.K	+3.22315865	0.027864
Myosin-7	P12883	R.LEEAGGATSVQIEMNK.K	+3.22315865	0.027864
Dihydropyrimidinase-related protein 2	Q16555	R.DIGAIAQVHAENGDIIAEEQQR.I	+3.223157523	0.027864
ADP-ribosylation factor 5	P84085	R.HYFQNTQGLIFVVDSNDR.E	+3.223158596	0.027864
ATP synthase subunit alpha	P25705	K.LEPS.K	+3.22315836	0.027864
ATP synthase subunit alpha	P25705	R.NVQAEEMVEFSSGLK.G	+3.223158377	0.027864
NADH dehydrogenase	O95298	K.TYGEIFEKF.H	+3.22315853	0.027864
Filamin-A	P21333	G.LEPS.G	+3.22315836	0.027864
Filamin-A	P21333	I.PFT.I	+3.22315836	0.027864
Parathymosin	P20962	K.SVEAAAELSAK.D	+3.223158229	0.027864
Myosin-8	P13535	K.NDLQLQVQSEADSLADAEER.C	+3.22315843	0.027864
Myosin-8	P13535	K.EKNDLQLQVQSEADSLADAEER.C	+3.223158613	0.027864
Tubulin beta-3 chain (TUBB)	Q13509	R.ISEQFTAMFR.R	+3.223158132	0.027864
Tubulin beta-4B chain (TUBB)	P68371	R.ISEQFTAMFR.R	+3.223158132	0.027864
Tubulin beta-2A chain (TUBB)	Q13885	R.ISEQFTAMFR.R	+3.223158132	0.027864
Tubulin beta chain (TUBB)	P07437	R.ISEQFTAMFR.R	+3.223158132	0.027864
Tubulin beta-4A chain (TUBB)	P04350	R.ISEQFTAMFR.R	+3.223158132	0.027864
Tubulin beta-2B chain (TUBB)	Q9BVA1	R.ISEQFTAMFR.R	+3.223158132	0.027864
Hemoglobin subunit alpha	P69905	L.ASVSTVLTSK.Y	+3.223158483	0.027864
Alpha-2-antiplasmin	P08697	R.QLTSGP.N	+3.223157523	0.027864
Fibrinogen alpha chain (FGA)	P02671	A.DSGEGDFLAEGGGVRGPR.V	+3.223158996	0.027864
Complement C4-A (C4A)	P0C0L4	R.NGFKSHALQLNN.R	+3.223158111	0.027864
Complement C4-A (C4A)	P0C0L4	R.NGFKSHAL.Q	+3.223158539	0.027864
Complement C4-A (C4A)	P0C0L4	H.ALQLNNRQI.R	+3.223158413	0.027864
Apolipoprotein L1	O14791	A.PFT.E	+3.22315836	0.027864
Collagen alpha-1	P20908	S.PSEI.G	+3.223158274	0.027864
Complement C3	P01024	R.IHWESASLL.R	+3.223158692	0.027864
Titin	Q8WZ42	S.KATL.F	-7.256112727	0.007996
Titin	Q8WZ42	P.PPPT.T	-2.186526157	0.048995
Titin	Q8WZ42	D.PSEI.L	+3.223158274	0.027864
Titin	Q8WZ42	K.LEPS.Q	+3.22315836	0.027864
Titin	Q8WZ42	R.TTLK.V	+3.223158464	0.027864
Titin	Q8WZ42	E.YAPP.K	-3.776166378	0.032523
Titin (EC 2.7.11.1)	Q8WZ42	I.ELSP.S	+3.223158437	0.027864
Titin (EC 2.7.11.1)	Q8WZ42	A.PFT.Y	3.22315836	0.027864
PDZ and LIM domain protein 1	O00151	M.PFT.A	3.22315836	0.027864

*The statistical significance level was set at P < 0.05.

These differentially expressed peptides’ C- and N-terminal cleavage sites were examined in the research. It’s interesting to note that these peptides mostly had four cleavage sites: the N-terminal amino acid of the detected peptide, the C-terminal amino acid of the identified peptide, and the N-terminal amino acid of the next peptide. Notably, Leucine (L) and Lysine (K) were found to be the most prevalent amino acids at the N-terminus of the detected peptide, whereas Arginine (R) was revealed to become the most abundant amino acid present at the C-terminus of the preceding peptide. Additionally, it was discovered that arginine (R) was the most prevalent amino acid at both the C-terminus of the detected peptide and the N-terminus of the next peptide (**Figure 2G**).

### 3.3 Bioinformatics analysis

**Figure 3:**
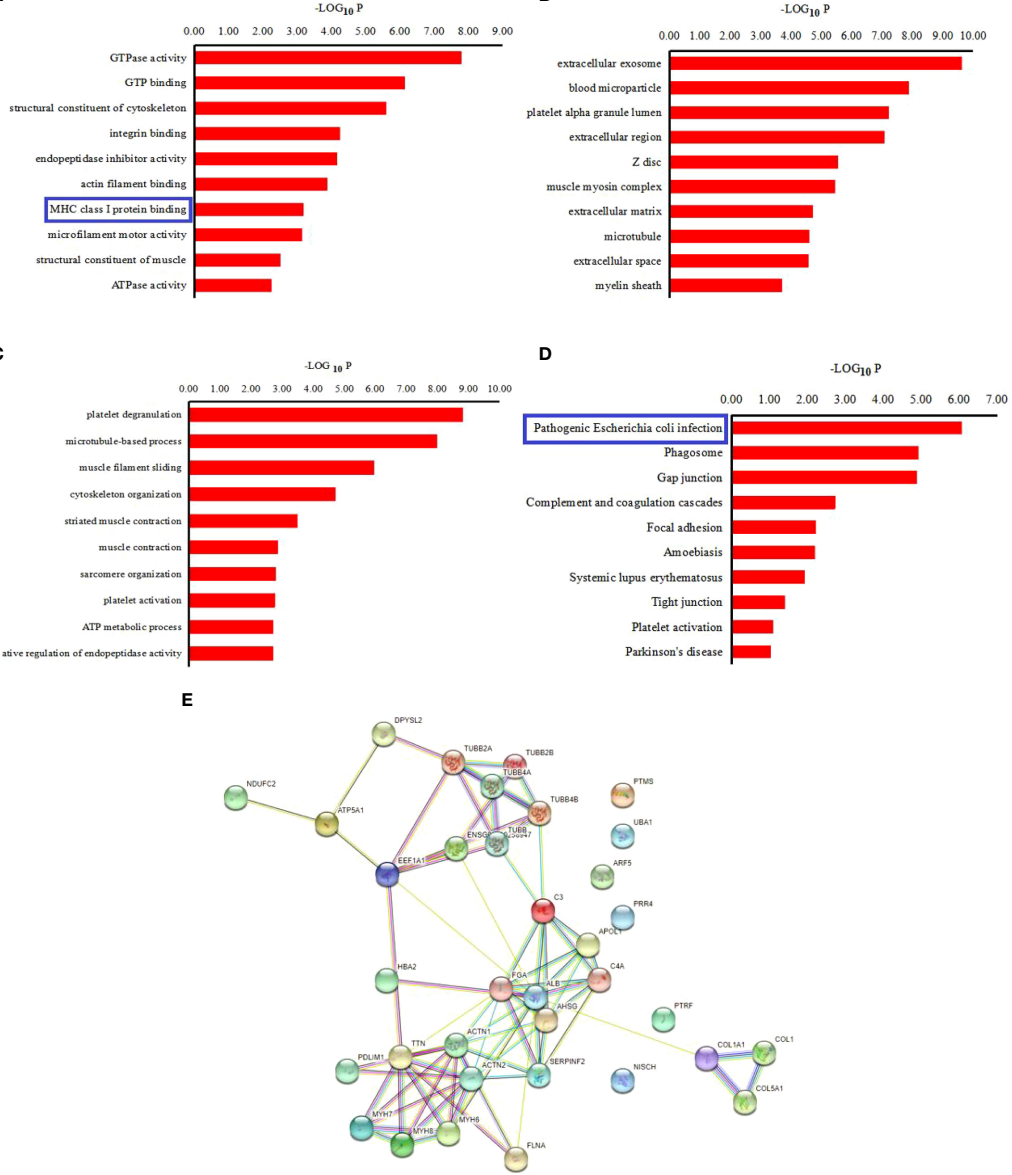
Bioinformatics analysis of pepetide precursor proteins: Gene ontology of 9 **(A)** Biological process of differentially expressed peptides precursor proteins. **(B)** Cellular components of differentialy expressed peptides precursor proteins. **(C)** Molecular function of differentially expressed peptides precursor proteins. **(D)** KEGG pathway analysis of the precursor proteins of the differentially expressed peptides. **(E)** Interaction network of precursor proteins of the differentially expressed peptides as determined with STRING (https://string-db.org/, version: 11.0). The confidence level: medium confidence 0.400.

The precursor proteins of these peptides were analyzed using the GO and KEGG pathways in the current investigation, to predict their potential functions. The results for GO analysis revealed enrichment of 10 ‘Molecular function’ categories for these peptides, which included “GTPase activity”, “GTP binding”, “structural constituent of cytoskeleton”, “Major histocompatibility complex (MHC) class I protein binding”, et al. (**Figure 3A**). Among of these, the “MHC class I protein binding” has been proved to be relevant to the occurrence of AS. The most enriched ‘cellular component’ categories included “extracellular exosome”, “blood microparticle”, “platelet alpha granule lumen”, et al. (**Figure 3B**). For these precursors, the ‘Biological function’ categories were mainly associated with “platelet degranulation”, “microtubule‐based process”, “muscle filament sliding”, “cytoskeleton organization”, “striated muscle contraction”, “muscle contraction”, “sarcomere organization”, “platelet activation”, “ATP metabolic process”, and “negative regulation of endopeptidase activity” (**Figure 3C**).

Additionally, the KEGG pathway analysis revealed that the precursor proteins were mostly linked to “Pathogenic Escherichia coli infection”, “Phagosome”, “Gap junction”, et al. (**Figure 3D**). For the “pathogenic E. coli infection” signal pathway, some inflammatory factors that affect the process of AS, such as TNF α, IL-1 β, IL-1, IL-8 as well as NF-κB, are associated with this signal pathway. The STRING website was used to further examine the interaction network of these peptide precursor proteins. **Figure 3E** shows an illustration of a typical STRING network interaction.

### 3.4 Screening and synthesis of peptides

3 peptides from the differential peptides based on the activity fraction of peptide ranker, difference multiple, and P-value in this study were screened out. The FGA-peptide (sequences: DSGEGDFLAEGGGVRGPR), C4A-peptide (sequences: NGFKSHAL), and TUBB-peptide (sequences: ISEQFTAMFR) which be named according to their precursor proteins were screened out in this experiment. **Table 3** displays the peptides’ chemical and physical attributes.

**Table 3:** The physical and chemical properties of 3 differentially peptides.

Precursor protein	Peptide segment	Peptide ranker	Number of amino acids	Molecular weight	Theoretical PI	The instability index	Aliphatic index	Grand average of hydropathicity
FGA	DSGEGDFLAEGGGVRGPR	0.723607	18	1775.85	4.32	143.52	25.79	-1.974
C4A	NGFKSHAL	0.323976	8	872.98	8.76	-28.82	61.25	-0.425
TUBB	ISEQFTAMFR	0.656122	10	1229.42	6.00	39.98	49.00	0.080

### 3.5 Cell viability analysis

#### 3.5.1 Cell culture

The cultured ligament fibroblasts, obtained from AS patients, were found/observed to be long and branched. Among these, few cells were flat and polygonal, with round or oval nucleus, and exhibited adherent growth patterns. When the bottom of the bottle became full, these fibroblasts got arranged in polar, indigo vortex, or radial shape, and the size of the cell body was observed to be the same.

#### 3.5.2 CCK‐8 assay

**Figure 4:**
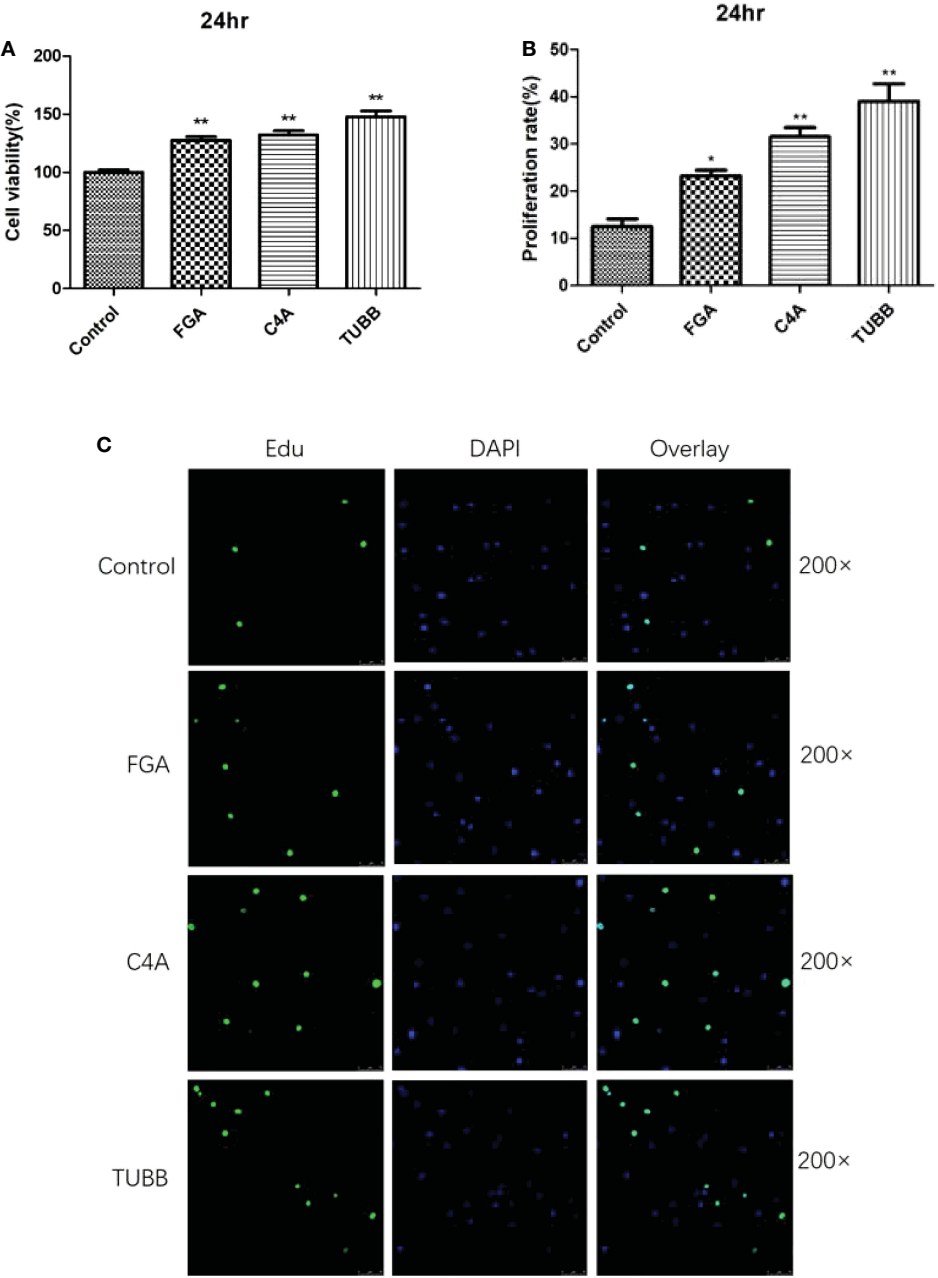
The results of Cell viability analysis verification by CCK8 and Edu staining: **(A)** The results of CCK8. **(B)** The results of Edu staining. **(C)** The 200x laser confocal microscope and photograph of Edu staining. * and ** indicates significant difference (*P* < 0.05) extremely significant difference (*P* < 0.01) compared with the control group.

Additionally, a CCK-8 test was used to confirm the impact of 3 differentially expressed peptides, namely FGA-peptide, C4A-peptide, and TUBB-peptide, on cell viability. The results for the assay showed that these three differentially expressed peptides could significantly promote the rate of viability in fibroblasts at 24 hr (P < 0.05). Among of these, TUBB-peptide was the most significant difference of the rate of cell viability (%) (P less than 0.01) (**Figure 4A**).

#### 3.5.3 Edu staining assay

The results for Edu staining showed that these three differentially expressed peptides could significantly promote the proliferation of fibroblasts at 24 hr, as compared to the control group (P<0.05). The CCK8 assay’s findings and these results were in agreement (**Figures 4B, C**).

These results revealed that FGA-peptide (peptide sequences: DSGEGDFLAEGGGVRGPR), C4A-peptide (peptide sequences: NGFKSHAL), and TUBB-peptide (peptide sequences: ISEQFTAMFR) could significantly promote the proliferation of fibroblasts *in vitro*, which might be highly relevant to the occurrence and development of AS.

## 4 Discussion

As an immune-mediated chronic inflammatory disease, AS is the most prevalent type of spinal arthritis worldwide, occurring at a high rate. AS is characterized by persistent inflammation of the spinal joints as well as attachment sites along with spinal fusion due to he formation of new bone (20). The etiological mechanism of AS is complex and develops through the integration of genetic, microbial, environmental and immune factors (21–23). Peptidomystomics was used in the current work to thoroughly investigate alterations in the peptide profiles of AS patients, and differently expressed peptides were effectively found in the patients’ plasma samples. The development of AS diagnosis and therapy will be aided by the possible bioactive peptides found in our investigation. The differently expressed peptides’ physicochemical characteristics & bioinformatics research provided a fresh perspective on AS’s mechanisms.

52 differentially expressed peptides that showed a change of more than 2 fold were found in this investigation. These peptides usually had 25 or fewer amino acids, and their molecular weights (MWs) were less than 3.0 kDa, indicating the validity of the peptides discovered in the current investigation. The same precursor protein served as the source of several of these peptides. Proteases are crucial in the process of protein cleavage, which produces the majority of peptides and necessitates the precise identification of cleavage sites. Importantly, the biological function of the resultant cleaved peptides would be greatly influenced by the proteases’ identification of various cleavage sites (24). The process of protein cleavage by proteases often adheres to a set of guidelines. The choice of candidate peptides is aided by physicochemical characteristics including peptide length, MW, PI, and cleavage sites.

The results for GO analysis revealed that ‘MHC class I protein binding’ was one of the 10 leading categories under “Molecular function” that were found to be highly enriched with regard to these peptides. This suggested that MHC class I protein was one of the proteins that were associated with AS. These outcomes matched the results of Wang et al. (25) and Watad et al. (26). MHC Class I molecules are important for the initiation and propagation of immune responses (27, 28). AS is a chronic, progressive inflammatory diseases, which might result in the MHC class I presentation of viral peptides and is a chronic infection that finally clears up or persists for the whole life of the host (29). The “Pathogenic Escherichia coli infection” route was mostly linked to the precursor proteins, according to the KEGG pathway analysis. Importantly, among immune-mediated inflammatory illnesses, ankylosing spondylitis (AS), psoriatic arthritis (PsA), psoriasis, inflammatory bowel disease (IBD), and noninfectious uveitis form a separate category (30, 31). Interestingly, the “Pathogenic Escherichia coli infection” pathway might be associated with these immune-mediated inflammatory diseases, especially AS and IBD and genetic factors in these two diseases might also be related. Ergin et al. found that the E. coli-specific Th1 response was significantly reduced in Crohn’s patients and to a lower extent also in AS patients (32), and Syrbe et al. found that the high frequency and enrichment of E coli-specific CD4 T cells in the inflamed joints of patients with AS (33), which suggested that the “Pathogenic Escherichia coli infection” might be relevant to AS. For the “Pathogenic Escherichia coli infection” pathway, the study recognized some important inflammatory factors, such as TNF-α, IL‐1β, IL‐1, IL‐8, and NF‐κb, which were associated with different signaling pathways. These factors might affect the progression of the disease (31).

The most prevalent cell type in connective tissue, fibroblasts, are primarily in charge of producing and transforming the extracellular matrix, which contains a high concentration of collagen as well as other macromolecules (34). They exhibit osteogenic traits and could develop into osteoblasts that are crucial for pathological heterotopic ossification & wound healing (35). The majority of research has demonstrated that fibroblasts act as the starting point for AS ligament ossification (36). Thus, identifying target molecules that prevent fibroblasts from diffusing into osteoblasts could serve as a theoretical foundation for the therapy of AS, which is extremely crucial for enhancing the prognosis of AS patients. Numerous studies have concentrated on the various signaling pathways engaged in the ectopic ossification of AS (37), but there have been relatively few investigations on peptideomics evaluation in relation to this process. This study further screened out 3 up‐regulated peptides from these differentially expressed peptides, according to activity fraction of peptide ranker, difference multiple, and P‐value. These three peptides included FGA-peptide (sequence “DSGEGDFLAEGGGVRGPR”), C4A-peptide (sequence “NGFKSHAL”), and TUBB-peptide (sequence ISEQFTAMFR). These three synthesized peptides were added to the fibroblasts derived from the patients with AS. Further tests to confirm the impact of these differentially expressed peptides included CCK-8 and Edu staining experiments. Synovial cell secretion could induce differentiation of fibroblasts into osteoblasts in ligaments, which might be one of the reasons for new bone formation in AS. Fibroblasts present in the granulation tissue of subchondral bone of hip joint and sacroiliac joint in AS patients proliferate abnormally, such that granulation tissue forms bone *via* the process of endochondral ossification, resulting in joint ossification and ankylosis (38). The results of the present study further revealed that these three differentially expressed peptides could significantly promote the proliferation of fibroblasts *in vitro* and verified the results of peptide identification. These results further suggested these peptides might be highly relevant for the occurrence and development of AS, provide new ideas for the prevention and treatment of as.

Precursor protein-encoding FGA-peptide (sequences: DSGEGDFLAEGGGVRGPR) is Fibrinogen alpha chain which associated with vascular endothelial growth factor (VEGF). FGA may activate the VEGFA-VEGFR2-FAK signalling pathway to promote angiogenesis (39). VEGF is a crucial regulator of angiogenesis, inflammation, vascular permeability, as well as tissue repair (40).Vascular endothelial growth factor (VEGF), which is typically elevated in axial spondyloarthritis, is associated to coagulation and fibrinolysis (axSpA) (41). VEGF plays a significant role in bone repair and regeneration by affecting angiogenesis and inflammation (42). Additionally, it has been mentioned as a predictive biomarker for axSpA, with greater VEGF levels being linked to an increased risk for the disease’s radiographic progression (43). Ankylosing spondylitis, as a type of axSpA, characterized by the sacroiliac joint’s spinal development on radiographs, the levels of VEGF can be elevated ≥600 pg/mL (41). Thus, in the current research *in vitro*, the FGA-peptide was speculated. FGA-peptide could increase the proliferation of fibroblasts with AS may contribute to inflammatory processes of AS by activating the VEGFA-VEGFR2-FAK signalling pathway.

Precursor protein-encoding C4A-peptide (sequence: NGFKSHAL) is Complement C4A. The complement system’s traditional route includes C4A. Systemic lupus erythematosus and type 1 diabetes are both linked to C4A deficiency, whereas schizophrenia and bipolar illness are linked to C4A overexpression (44). An essential component of humoral and innate immunity is the complement system. Inhibiting the complement system in an animal model of AS might enhance therapy for the condition (45). Patients with systemic sclerosis, a musculoskeletal condition, have an active complement system (46). Ji-Hyun Lee et al. (47) has identified C4A as a potential biomarker for AS. Thus, we supposed that C4A-peptide might be related to the progress of AS by affecting the complement system.

Among these 3 peptides, the TUBB-peptide (sequence: ISEQFTAMFR) was associated with the most significant difference in cell proliferation. Precursor protein-encoding TUBB-peptide forms a dimer with α‐tubulin and functions as a microtubule’s structural component. TUBB serves as an aprotein-coding gene, which encodes for β‐tubulin protein. Diseases associated with TUBB include cortical dysplasia, complex brain malformations, skin Creases, and congenital symmetric circumferential (48). GTP binding and structural molecular activity are two Gene Ontology (GO) annotations associated with TUBB. Signaling pathways related to TUBB include the development Slit‐Robo signaling and the innate immune system (49). In a previous study, Chang et al. (50) revealed that TUBB exhibited higher expression in the synovial membranes of patients with rheumatoid arthritis. In fact, TUBB has been reported to be one of the differentially expressed genes in rheumatoid arthritis (51). TUBB might be associated with autoimmune diseases. In the present study, one of the peptides with differential expression in the plasma of AS patients was found to be the TUBB-peptide. It significantly increased the proliferation of fibroblasts *in vitro*. Therefore, the study conjectured that TUBB-peptide might be highly relevant for the occurrence and development of AS by influencing the Slit‐Robo signaling pathway.

## 5 Conclusion

The present study reported the use of peptidomics for the first time for the analysis of the peptides present in the plasma of AS patients. The study identified 52 differentially expressed peptides using mass spectrometry. Further bioinformatics research revealed that these differently produced peptides maybe associated with “MHC class I protein binding” and “Pathogenic Escherichia coli infection” pathways, which might affect the progression of AS. Cell viability analysis verified the results of peptide identification and identified 3 peptides (sequence: DSGEGDFLAEGGGVRGPR, NGFKSHAL, ISEQFTAMFR) which might be highly relevant for the occurrence and development of AS, and may enhance this dangerous disease’s clinical results. Overall, this research might provide fresh perspectives on the AS molecular mechanisms based on peptide omics.

It should be noted that this study has certain limitations. Firstly, due to the relatively small sample size, further data collection is still needed in future research. Secondly, the mechanism of the relationship between these differentially expressed peptides and AS would be explored in the future. Further cell function research, signal pathway research and animal experimental research will be carried out according to the results obtained.

## Data availability statement

The original contributions presented in the study are included in the article/Supplementary Material. Further inquiries can be directed to the corresponding author.

## Ethics statement

The studies involving human participants were reviewed and approved by The Ethics Committee of Tongren hospital. The patients/participants provided their written informed consent to participate in this study.

## Author contributions

Collection of clinical data, Y-JX. Performed the experimental work and Cell viability analysis verification, G-NZ and LJ. Critically evaluated the study, LJ. Drafted the manuscript, G-NZ. Revision and final approval of the manuscript, LJ. All authors contributed to the article and approved the submitted version.
